# Synchronous parathyroid adenoma and thyroid papillary carcinoma: a case report

**DOI:** 10.1186/1757-1626-2-9121

**Published:** 2009-11-30

**Authors:** Ioannis P Iakovou, Iordanis E Konstantinidis, Alexandra I Chrisoulidou, Argyrios S Doumas

**Affiliations:** 1 3rd Academic Nuclear Department Papageorgiou Hospital, Thessaloniki, Greece; 2 2nd Academic ORL Department Papageorgiou Hospital, Thessaloniki, Greece; 3 Endocrinology Department, Theageneio Anticancer Institute, Thessaloniki, Greece

## Abstract

A 51-year-old female patient presented with atypical chest pain, laryngo-oesophageal reflux, increased levels of serum calcium and parathyroid hormone. Ultrasonography showed a multinodular goiter with a prominent solid nodule in the lower left thyroid lobe and a solid hypoechoic nodule outside this area.

Tc99m-sestamibi parathyroid scintigraphy was performed to investigate a primary hyperparathyroidism, revealing an area with increased uptake in the lower left thyroid lobe and another area with marked uptake lower than this level. Thyroid scintigraphy with 99mTc showed a cold nodule of the left lower pole. FNA of the thyroid nodule was positive for papillary carcinoma later verified by postoperative histopathology.

This case underlines the need for a clinical high index of suspicion for synchronous hyperparathyroidism and thyroid cancer.

## Case Report

A 51-year-old female patient presented with symptoms of atypical chest pain and laryngo-oesophageal reflux. The patient had a past medical history of nephrolithiasis with normal renal function. Blood tests revealed increased levels of serum calcium of 12.8 mg/dL (normal, 9.0-10.6 mg/dL), incrased levels of parathyroid hormone (PTH) at 115 pg/mL (normal, 12-72 pg/mL), and a low serum phosphorus level of 2.4 mg/dL (normal, 2.5-4.5 mg/dL), findings suggestive for primary hyperparathyroidism. Free thyroxine (FT4), triiodothyronine (FT3) and thyroid-stimulating hormone (TSH) levels were within normal limits.

Ultrasonography displayed a multinodular goiter (total thyroid volume was about 43 ml) with a prominent solid nodule in the lower third of the left lobe of the thyroid gland and an additional solid hypoechoic nodule outside the gland but in close relation to the lower pole of the left lobe of it. Multiple endocrine neoplasia (MEN) syndrome was excluded since no blood test no abdomen CT gave evidence of pheochromocytoma existence.

**Figure 1:**
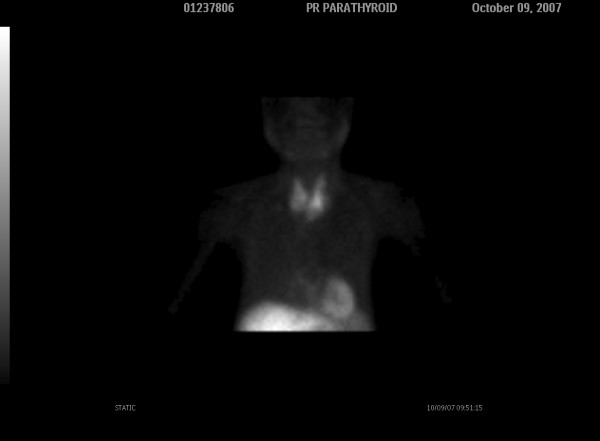
**The early scan (15 minutes after the injection of 20 mCi ^99m^Tc-sestamibi) shows intense focal uptake in the inferior aspect of the left lobe (white arrow) and faint retention lower than the left lobe of the thyroid gland**.

**Figure 2:**
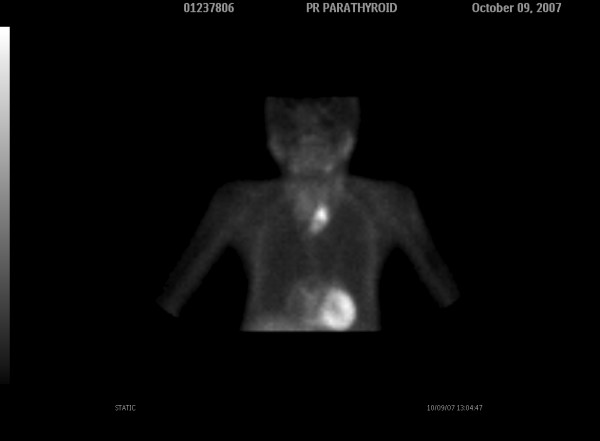
**Delayed image (3 hours later) shows the focal 99mTcSestamibi retention in the left lobe and clearly a synchronous faint retention lower than its lower end (white arrow)**.

Dual phase 99mTc-sestamibi parathyroid scintigraphy was performed in order to investigate the etiology of primary hyperparathyroidism. The early images obtained 15 minute after 20 mCi radiotracer injection, revealed an area with increased radioactivity in the left lower thyroid lobe (Figure 1) and a faint radioactivity lower than this level. The delayed image obtained 3 hours after injection showed persistent activity in the left lower thyroid lobe and more clearly the faint radioactivity lower than the level of the left thyroid lobe (Figure 2).

**Figure 3:**
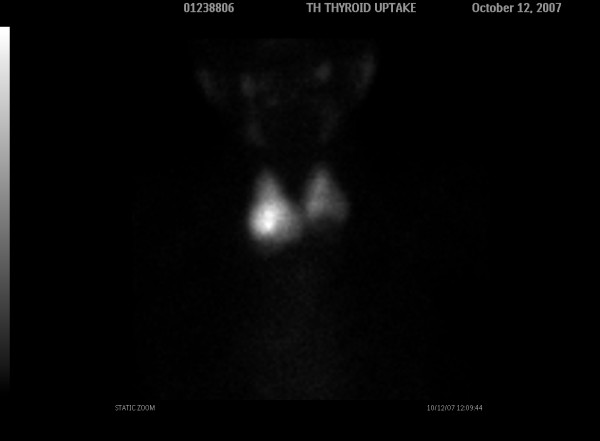
**Thyroid scan with 99mTc 2 days later shows a cold nodule in the lower end of the left lobe (white arrow)**.

Two days later a thyroid scintigraphy with 99mTc was performed for thyroid pathology investigation in which a cold nodule of the left lower pole was detected (Figure 3).

The patient underwent parathyroidectomy and a total thyroidectomy. No palpable cervical lymph nodes were discovered after detailed intraoperative exploration of the region. Histopathology results revealed parathyroid adenoma (1.5 cm greater diameter) in the left lower parathyroid gland and coexistent papillary carcinoma of the left lower lobe of thyroid gland (infiltrating, 2 cm in greater diameter).

The patient's postoperative course was uneventful, normocalcemia was achieved immediately after parathyroidectomy, and she was discharged from the hospital on the third postoperative day. Fifty days later the patient received 100 mCi ^131^I to ablate any residual thyroid parenchyma followed by treatment with suppressive doses of thyroxine (T4; 175 mg/day).

## Discussion

Primary hyperparathyroidism is a rare condition, however its incidence presents a noticeable increase over the last decades [1].

An association between hyperparathyroidism and well-differentiated thyroid carcinoma has been reported in many studies. In a retrospective study of 824 patients who had undergone cervical exploration for hyperparathyroidism in which at least a thyroid lobectomy was carried out, thyroid carcinoma was detected in 18 patients (8.6%) with primary hyperparathyroidism [2].

Burmeister et al additionally compared the incidence of thyroid carcinoma among groups of primary, secondary and tertiary hyperparathyroidism patients and found that the prevalence of thyroid carcinoma in a population of patients undergoing parathyroidectomy for hyperparathyroidism was the same irrespective of whether the patients had primary, secondary or tertiary hyperparathyroidism [2]. They concluded that the detection of thyroid carcinoma at the time of parathyroidectomy is related to risk factors that are probably not associated with hyperparathyroidism. The association between thyroid carcinoma and hyperparathyroidism may be coincidental.

Diagnostic procedure for the preoperative localization of a suspected parathyroid adenoma includes a 99mTc sestamibi scintigraphy. Taillefer et al developed a double-phase imaging procedure with 99mTc sestamibi for the detection and localization of parathyroid adenomas based on the fact that 99mTc sestamibi washes out more rapidly from the thyroid than from abnormal parathyroid tissue [3]. The above procedure improved surgeons' ability to localize parathyroid adenomas preoperatively [4].

The presence of a parathyroid adenoma is defined as a focal area showing increased 99mTc-sestamibi uptake in projection of the thyroid bed and surrounding areas including mediastinum. This area presents either a relatively progressive increase over time or a fixed uptake persisted on delayed imaging. On the contrary the uptake in the surrounding normal thyroid tissue, progressively decreases over time. The sensitivity of the dual-phase protocol varies and is influenced by numerous factors. These factors are related to the tumour such as tumoral tissue volume, oxyphilic cell content, P-glycoprotein expression, mitochondrial structure or related to the procedure set up such as the dose of radiotracer, the timing of imaging after radiotracer injection, and equipment and settings [5,6].

However, sestamibi scan may be positive in different benign and malignant thyroid tumours [7]. This is because the tracer is a lipophilic cationic molecule concentrating in cells with different metabolic background with active or passive transport and intramithochondrial sequestration. Thus, ambiguous sestamibi scan images may be observed when parathyroid and thyroid tumours are associated. This underscores the need of an integrated approach using the combination of sestamibi scan with other imaging modalities (e.g. in our case thyroid scintigraphy) in the differential diagnosis of selected cases of primary hyperparathyroidism, especially when associated to multinodular thyroids, and/or after failure of previous parathyroid surgery.

## Conclusion

Certainly hyperparathyroidism should not be considered as a high suspicious condition for thyroid cancer; instead, each patient with hyperparathyroidism should be studied including as a possible part of a MEN syndrome while the presence of a non-toxic goiter with suspicious nodule should also be studied, including a FNAB.

Although the incidence of thyroid malignancy in patients with hyperparathyroidism is relatively low, delayed thyroid clearance of 99mTc sestamibi may need to be investigated further. This case underlines the need for a clinical high index of suspicion for synchronous hyperparathyroidism and thyroid cancer. Dual-phase 99mTc sestamibi parathyroid imaging may be useful in detecting thyroid cancer even along with other diagnostic modalities.

## Consent

Written informed consent was obtained from the patient for publication of this case report and accompanying image. A copy of the written consent is available for review by the Editor-in Chief of this journal.

## Competing interests

The authors declare that they have no competing interests.

## Authors' contributions

II was the major contributor in writing the manuscript. IK performed the clinical assessment and revised the manuscript. AC performed the literature review and contributed in writing the manuscript while AD gave final approval and revised the article. All authors read and approved the final manuscript.
